# Bevacizumab as a treatment option for choroidal neovascularisation due to large optic nerve drusen in a 14-year-old girl

**DOI:** 10.3205/oc000160

**Published:** 2020-08-06

**Authors:** Nils Alexander Steinhorst, Martin Spitzer, Christos Skevas

**Affiliations:** 1 Department of Ophthalmology, University Medical Center Hamburg-Eppendorf, Hamburg, Germany

**Keywords:** juvenile, optic disc drusen, choroidal neovascularisation, cystoid edema, bevacizumab

## Abstract

**Purpose:** To report the effects of a single intravitreous injection of bevacizumab for the treatment of secondary choroideal neovascularisation due to large optic disc drusen.

**Methods:** A 14-year-old female patient with painless loss of vision in one eye presented with unusually large optic disc drusen and juxtapapillary choroidal neovascularisation with subretinal hemorrhage. She was treated with a single intravitreous injection of bevacizumab.

**Results:** Visual acuity increased from 20/100 to 20/25 within 4 weeks after injection and remained at this level during the 12-month follow-up period.

**Conclusions:** Bevacizumab is a possible primary treatment option for secondary choroidal neovascularisation due to large optic disc drusen in children as an alternative to other more invasive or complex procedures.

## Introduction

Optic disc drusen (ODD) are remnants of mucopolysaccharides from degenerated ganglion cells which calcify and accumulate within the optic nerve head over time [1]. The incidence of ODD in children is reported to be 0.4%. They seem to become visible around the age of 12 [2].

With increasing size, they raise the optic disc, blur out the edges, and lead to abnormal vascular branching and formation of cilioretinal vessels, causing hemorrhages [3]. This destruction of the parapapillary anatomy can cause visual field defects and the formation of choroidal neovascular membranes (CNVM) that lead to decreased visual acuity (VA) [4].

Because of the rare occurrence of CNVM associated with ODD, different therapeutic approaches have been reported only via small case studies. Next to surgical removal [5] and photodynamic therapy [6] or laser coagulation [7], intravitreous injection of anti-VEGF agents seems to be an option with long-term satisfying results [8], [9], [10], [11].

## Patient and methods

**Figure 1:**
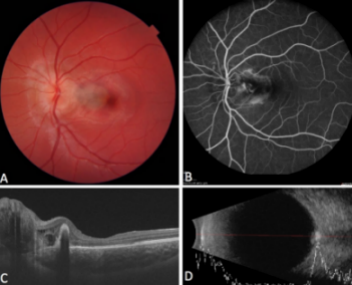
A) Funduscopy revealed a prominent and blurry optic disc with subretinal hemorrhage spreading towards the edge of the macula region; B) CNVM made visible through mid-phase IVFA; C) OCT scan with ODD and parapapillary cystoid edema, subretinal scarring; D) Shallow but prominent ODD are revealed through hyperreflectivity in low-gain mode using ultrasound.

A 14-year-old female patient presented at our clinic with painless loss of vision in her left eye, slowly progressing for over one week. VA had dropped to 20/100 on the affected side, whilst retaining 20/20 vision on the other eye. Intraocular pressure was within physiological limits. Further examination showed a blurry and prominent optic disc with an adjacent decent subretinal hemorrhage (Figure 1 (Fig. 1)). The contralateral optic disc also displayed a slight blurriness, but there was no sign of subretinal hemorrhage. Optical coherence tomography (OCT) showed the formation of a juxtapapillary cystoid macula edema (CME) with subretinal scarring. Corresponding to this, the intravenous fluorescein angiography (IVFA) displayed leakage at this location throughout all phases, followed by pooling. Additionally, ultrasound was carried out, which exposed unusual large and deeply located ODD on both eyes. These were however significantly larger and closer to the optic disc’s surface on the affected left eye.

After discussing the potential treatment options and risks with the child’s parents, a single intravitreous injection of 1.25 mg bevacizumab was given under general anesthesia.

## Results

**Figure 2:**
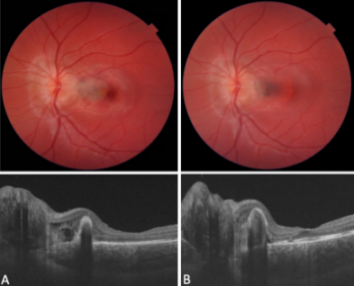
Fundus image and OCT scan A) prior to treatment and B) 12 months after injection. The subretinal hemorrhage and cystoid edema resolved, leaving behind a dissociation of pigment epithelium and a shallow subretinal scar.

VA increased to 20/25 over a period of 4 weeks. The cystoid edema as well as the subretinal hemorrhage resolved, leaving a small subretinal scar and a mild dissociation of the pigment epithelium (Figure 2 (Fig. 2)). The patient and her parents were instructed to perform self-tests using the Amsler grid and to report to the clinic, should any deterioration occur. Further appointments were scheduled regularly at an interval of 4 weeks for 6 months overall, then followed by examinations every 12 weeks. During this phase, the VA fluctuated between 20/25 and 20/20. There was no recurrence of CME or subretinal bleeding during the follow-up period.

## Discussion

In the last decade, intravitreal injections of anti-VEGF agents have proven to be an effective therapy for a variety of retinal and especially macula diseases, for example neovascular age-related degeneration and diabetic macular edema. Disease-adapted guidelines were promoted and published for the initial therapy as well as for the long-term intervention [11], [12], [13]. Because of the rare incidence of CNVM secondary to ODD, especially in children, there are no established or widely accepted treatment regimens until today.

In our case, the patient showed a complete remission after only one injection. The options of further injections at a fixed interval, or of close observation and treatment only if required were discussed with the patient and her parents. The decision was made towards short-interval follow-up examinations. Compared to the results of anti-VEGF therapy that have been published in other case series, the juvenile secondary CNVM in our case responded better to this form of treatment. Until today, no further injections were necessary, confirming our choice of treatment plan.

## Notes

### Competing interests

The authors declare that they have no competing interests.
